# The value of postmortem computed tomography in paediatric natural cause of death: a Dutch observational study

**DOI:** 10.1007/s00247-017-3911-0

**Published:** 2017-07-05

**Authors:** Rick R. van Rijn, Erik J. Beek, Elise M. van de Putte, Arianne H. Teeuw, Peter G. J. Nikkels, Wilma L. J. M. Duijst, Rutger-Jan A. Nievelstein, on behalf of the Dutch NODO Group

**Affiliations:** 1 Department of Radiology, Emma Children’s Hospital, Academic Medical Centre Amsterdam, Meibergdreef 9, 1105 AZ Amsterdam Zuid-Oost, the Netherlands; 2 Department of Radiology, Wilhelmina Children’s Hospital, University Medical Centre Utrecht, Utrecht, the Netherlands; 3 Department of Paediatrics, Wilhelmina Children’s Hospital, University Medical Centre Utrecht, Utrecht, the Netherlands; 4 Department of Paediatrics, Emma Children’s Hospital, Academic Medical Center Amsterdam, Amsterdam, the Netherlands; 5 Department of Pathology, Wilhelmina Children’s Hospital, University Medical Centre Utrecht, Utrecht, the Netherlands; 6 Dutch Forensic Medical Association, Rotterdam, the Netherlands

**Keywords:** Autopsy, Cause of death, Children, Computed tomography, Postmortem, Sudden death

## Abstract

**Background:**

Postmortem CT is a relatively new field of interest within paediatric radiology. This paper focusses on its value in cases of unexpected natural death.

**Objective:**

We report on an observational Dutch study regarding the value of postmortem CT in children with an assumed natural unexpected death because postmortem CT is part of the Dutch NODO (additional investigations of cause of death) procedure.

**Materials and methods:**

We included consecutive children who fulfilled criteria for the NODO procedure and were therefore referred to one of the centres for the procedure. Postmortem CT was performed in all cases and skeletal survey was performed in all children ages <5 years. The cause of death was defined in a consensus meeting.

**Results:**

We included a total of 54 children (30 boys, median age 1.1 years, and 24 girls, median age 0.8 years). A definitive cause of death was established in 38 cases. In 7 cases the cause of death could be identified on postmortem CT. In 7 cases imaging findings were clinically relevant but did not lead to a cause of death. In the remaining 40 cases postmortem CT did not add to the diagnostic workup.

**Conclusion:**

Our study shows that in a group of children who unexpectedly died of an assumed natural cause of death and in whom a cause of death was found at autopsy, postmortem CT detected the cause of death in a minority of cases (12.9%). In the majority of cases (74.1%) postmortem CT did not add value in diagnosing the cause of death.

**Electronic supplementary material:**

The online version of this article (doi:10.1007/s00247-017-3911-0) contains supplementary material, which is available to authorized users.

## Introduction

In 2013, in a population of 3,870,773 inhabitants younger than 20, 1,112 persons (0.29 per 1 million) died in the Netherlands [1]. Of the children who died in 2014, the majority (*n*=645) were younger than 1 year. These figures show that there is a low risk of death before the age of 20 years in the Netherlands. But in these rare cases the cause of death should be thoroughly investigated to prevent further cases of early demise in case of genetic-based diseases or disorders. Furthermore, determining cause of death can help parents in overcoming the grief of losing their child [2].

In the last decades there has been increasing interest in the use of postmortem radiology, especially postmortem CT and postmortem MRI, as an adjunct to the conventional autopsy. Most studies have been performed in foetuses and neonates and almost exclusively in hospitals or forensic populations [3–8]. Little is known about the value of postmortem CT in a paediatric population in whom a natural cause of death is assumed. A change in Dutch law in October 2010, whereby consultation with a municipal coroner became mandatory in all paediatric deaths (Article 10A of the Burial Act), led to the implementation of the so-called NODO procedure [9]. This is a procedure in which in case of an assumed natural unexpected death a thorough postmortem evaluation, including postmortem radiology, is offered to the parents of the deceased child. The NODO procedure consists of a stepwise approach under the guidance of a forensic physician and a paediatrician, including a home visit by the forensic physician to assess the circumstances where the body was found, a full medical and medico–social history, an external examination, radiologic examination (conventional radiography in children ages <5 years, and postmortem CT in all cases), laboratory examination, genetic testing and conventional autopsy. The last step of the NODO procedure, the conventional autopsy, required consent from both parents during the time of the study. If during the NODO procedure an indication of a non-natural cause arises the procedure is aborted and a legal investigation is initiated. The findings of this stepwise procedure are discussed in a consensus meeting in which all relevant medical disciplines participate and during this meeting a final cause of death, if possible, is determined. A more in-depth presentation of the NODO procedure, including the legal implications, is presented in Appendix A.

The aim of this observational study was to assess the value of postmortem CT in diagnosing the cause of death in children with an assumed natural unexpected death.

## Materials and methods

### Patients

Between October 2012 and December 2013 two Dutch University Hospitals — the Emma Children’s Hospital – Academic Medical Center Amsterdam (AMC) and the Wilhelmina Children’s Hospital – University Medical Centre Utrecht (UMCU) — acted as national centres for the NODO procedure.

All consecutive children ages <18 years who fulfilled the criteria for the NODO procedure and who were referred to one of the two national centres were included in the study. In all cases, informed consent for the autopsy was requested by a trained paediatrician who explained the additional value of the autopsy. From this study population, those children who did not undergo postmortem radiologic examination and those for whom no permission for autopsy was given were excluded from the final data analysis because in these cases the reference standard was missing. We also excluded cases where during the NODO procedure an indication of a non-natural cause arose, the reason being that in these cases the final cause of death resulting from the forensic autopsy was unknown to us.

The internal review board of the AMC issued a waiver for retrospective anonymized chart reviews, therefore no approval was requested.

### Postmortem skeletal survey

The NODO protocol dictates that a skeletal survey be performed in all children ages <5 years, according to the guidelines of the Royal College of Radiologists and the Royal College of Paediatrics and Child Health [10].

For this study, the skeletal survey was scored independently by one of three paediatric radiologists (E.J.B. with 29 years of experience, R.-J.N. with 14 years of experience, and R.R.vR. with 12 years of experience). We used the reports of the initial skeletal survey.

### Postmortem computed tomography

A full-body postmortem CT was performed in all children. The protocol consisted of a scan of the head/neck (parameters of 120 kV, 285 mAs, 0.9-mm slice thickness, 0.45-mm increment, pitch 0.392, collimation 64 × 0.625, with bone and brain reconstructions) and chest/abdomen/extremities (parameters of 120 kV, 250 mAs, 3.0-mm slice thickness, 2.0-mm increment, pitch 0.983, collimation 64 × 0.625, with bone and soft-tissue reconstructions). CT scans were performed on a Philips Brilliance (64 multi-detector CT; Philips Medical Systems, Best, the Netherlands) at the AMC and a Philips Brilliance (16 multi-detector CT; Philips) at the UMCU.

During the NODO procedure the postmortem CT was reviewed by one of three experienced paediatric radiologists (the same as for the postmortem skeletal survey). For this study the scans were reviewed by two paediatric radiologists, one reviewer per centre (R.-J.N. and R.R.vR.). The radiologists did not have access to clinical data.

### Other data

In all cases a full clinical and social history was obtained, a full physical exam was performed, laboratory investigations including blood culture, basic haematological workup, renal/liver and pancreatic functions, and electrolytes were obtained. In select patients additional genetic, metabolic and virology testing was performed. The results of these tests were correlated with imaging in cases where clinically significant findings (e.g., cause of death) were found on imaging. A full analysis of the other medical tests performed in the NODO procedure is outside the scope of this study. The cause of death was obtained from the pathology reports that were finalised after a consensus meeting with all participating disciplines.

### Data analysis

The patients were grouped into two categories based on the autopsy outcome. The first category was composed of cases where the full autopsy procedure led to a definitive cause of death. This population was used to calculate sensitivity. The second category was composed of cases where the full autopsy procedure did not lead to a cause of death, including those with sudden infant death syndrome (SIDS) and sudden unexpected death in epilepsy (SUDEP). For this study these three categories were combined into one group.

## Results

### Patients

**Fig. 1:**
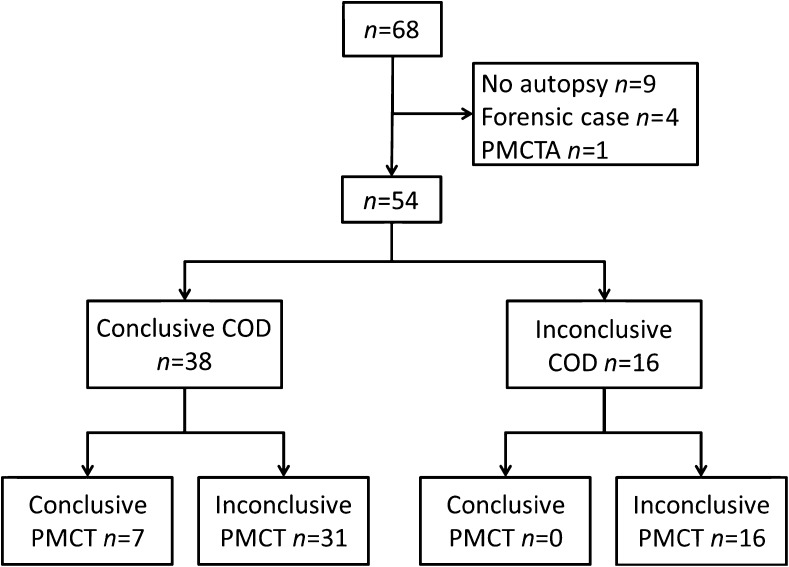
Standard for reporting of diagnostic studies (STARD) flowchart of cases referred for the NODO (additional investigations of cause of death) procedure. *COD* cause of death, *PMCT* postmortem computed tomography, *PMCTA* postmortem CT angiography

During the study period a total of 68 children were initially identified, which is significantly fewer than the actual number of children who, according to Dutch law, should have been included in the NODO procedure. The distribution over both centres was equal with 34 cases analysed in each centre. In 9 cases (13.2%, 5 boys and 4 girls) no permission for autopsy was obtained. In 4 cases (5.9%, all boys, ages 16 years 4 months, 10 years 5 months, 5 months, and 5 days) an indication of a non-natural cause arose during the NODO procedure and in these cases a legal investigation was started (Fig. 1). In two of these cases the radiologic examination led to the abortion of the NODO procedure; these included one case of an epidural hematoma and one case of what appeared on conventional radiography and CT to be a linear skull fracture (this case has been published; it actually turned out to be an accessory skull suture on autopsy) [11]. Because in these 13 cases either autopsy was not performed or the outcome was unknown to the author group, we excluded them from data analysis. In another case postmortem CT including postmortem CT angiography was performed at an outside hospital prior to transport to the NODO centre, and because postmortem CT angiography was not part of the routine protocol we also excluded this patient from final analysis.

After exclusions, we included a total of 54 children (79.4%, 30 boys and 24 girls) in the data analysis (Table 1).

**Table 1:** Patients included in the study

	Total			Conclusive cause of death			No conclusive cause of death		
	Total (*n*=54)	Boys (*n*=30)	Girls (*n*=24)	Total (*n*=38)	Boys (*n*=21)	Girls (*n*=17)	Total (*n*=16)	Boys (*n*=9)	Girls (*n*=7)
Median age	1.0 years	1.1 years	0.8 years	2.8 years	4.3 years	1.3 years	0.3 years	0.2 years	0.5 years
Age range	2 days – 17.9 years	5 days – 17.8 years	2 days – 17.9 years	2 days – 17.9 years	5 days – 17.8 years	2 days – 17.9 years	19 days – 15.1 years	0.1 years – 1.3 years	19 days – 15.1 years

Based on the full NODO procedure, a definitive cause of death was established in 38 cases, 21 boys and 17 girls, with a median age of 2.8 years (range 2 days to 17.9 years) (Tables 1 and 2). No definitive cause of death was established in 16 cases, 9 boys and 7 girls, with a median age of 0.3 years (range 19 days to 15.1 years). The causes of death are detailed in Table 2.

**Table 2:** Causes of death in all 54 patients after consensus meeting

	*n*	%
No cause of death found	16	29.6
Infection^a^	16	29.6
Cardiovascular^b^	11	20.3
Digestive tract^c^	4	7.4
Endocrine^d^	3	5.6
Neurological^e^	2	3.7
Pulmonary^f^	2	3.7

^a^Includes one patient with systemic infection with multi-organ failure, nine with bacterial infections and six with viral infections

^b^Includes one patient with Loeys–Dietz, five with congenital anomalies, three with a cardiomyopathy, one with cardiomyositis and one with a drug-induced myocardial infarction

^c^Includes one patient with intussusception and three with strangulation ileus

^d^Includes two patients with keto-acidosis and one with thyrotoxicosis

^e^Includes one patient with a drain dysfunction and one with an aqueduct anomaly

^f^Includes one patient with congenital pulmonary hypoplasia and one with massive pulmonary haemorrhage

### Postmortem skeletal survey

A full skeletal survey was performed in all 35 children ages <5 years. No clinically relevant findings were found in any of these children. Minor normal variants that were not clinically relevant were reported in four cases.

### Postmortem computed tomography

**Fig. 2:**
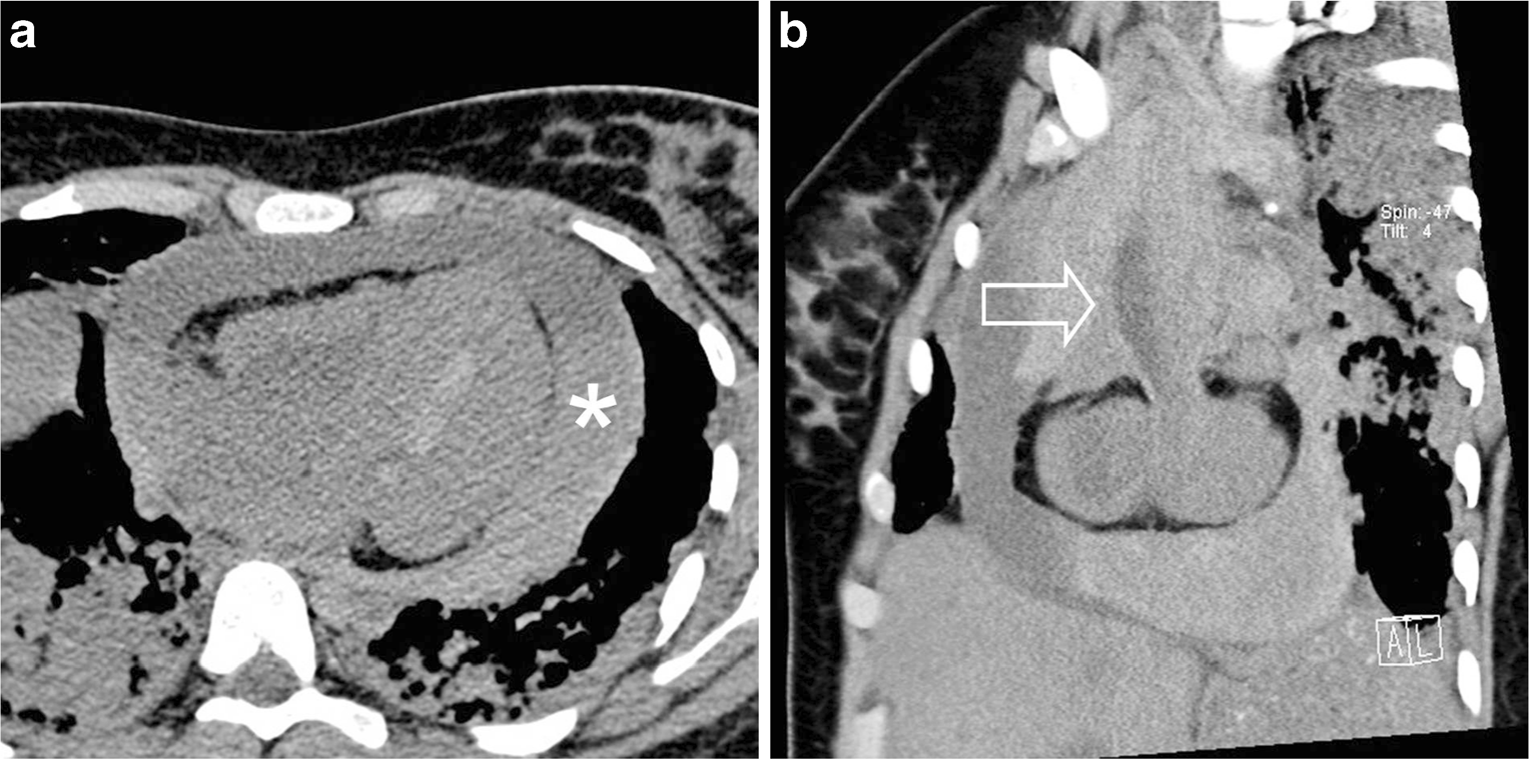
Cardiovascular cause of death. Postmortem CT in a girl age 17 years 2 months. **a** Axial image shows a haemopericardium (*asterisk*) with sedimentation of blood. **b** Sagittal oblique reconstruction shows a fusiform aortic aneurysm (*arrow*). Autopsy and genetic testing revealed a Loeys–Dietz syndrome

**Fig. 3:**
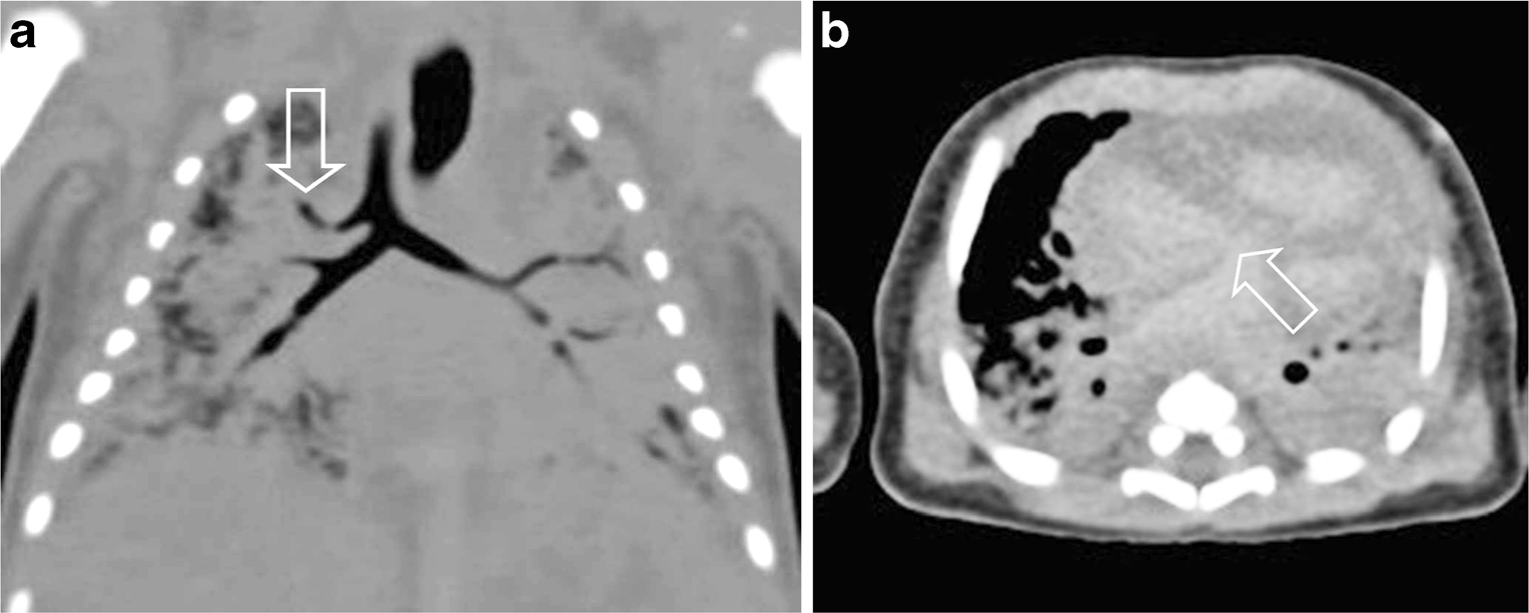
Cardiovascular cause of death. Postmortem CT in a 5-day-old boy. **a** Coronal reconstruction shows a tracheal right upper lobe bronchus (*arrow*). **b** Axial image shows an atrial septal defect (*arrow*). Both findings were confirmed at autopsy

**Fig. 4:**
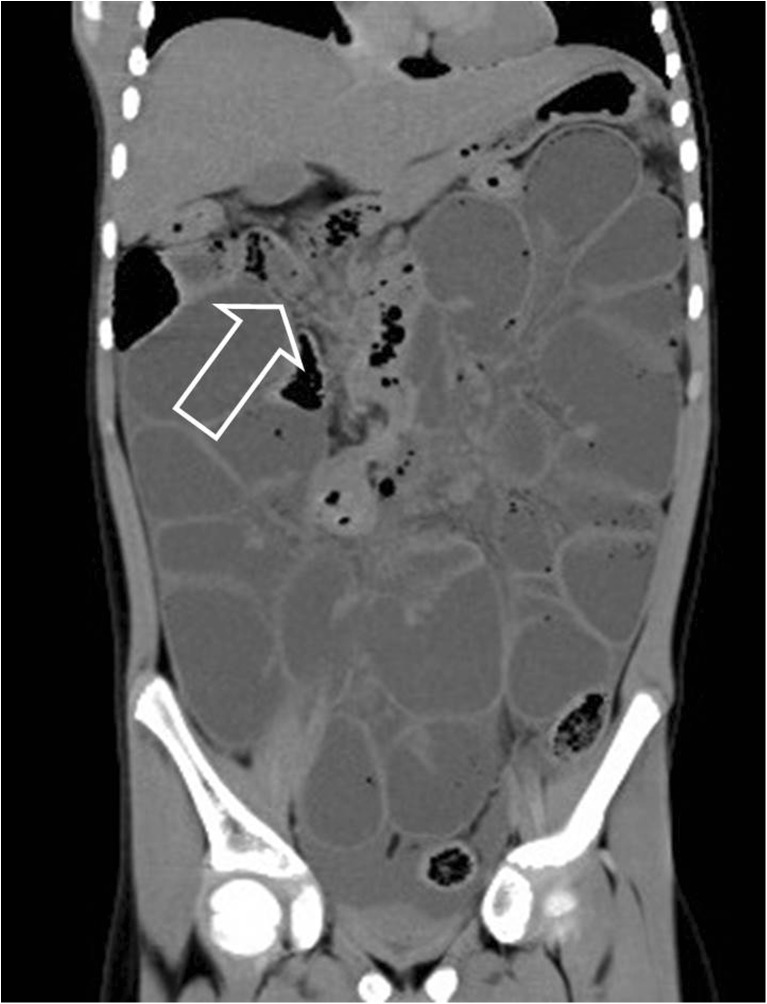
Postmortem CT in a girl age 6 years 11 months with an adhesion ileus. Coronal reconstruction shows the presence of collapsed small-bowel loops in the right upper abdomen (*arrow*). On autopsy an adhesion ileus was confirmed

**Fig. 5:**
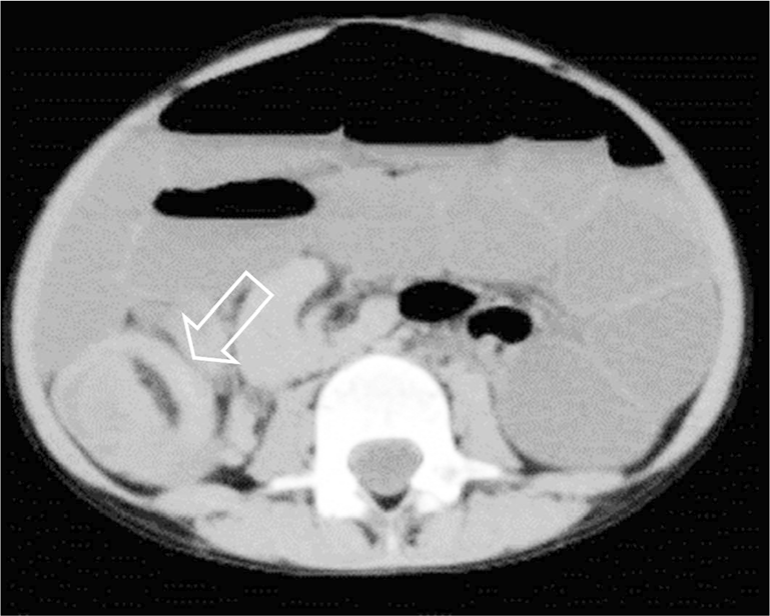
Axial postmortem CT in a boy age 4 years 4 months shows an ileocolic intussusception (*arrow*). At autopsy a Meckel diverticulum was found as a pathological lead point

Postmortem CT was performed in all included cases (*n* = 54), and CT identified a cause of death in 7/54 (12.9%) cases. Details of these cases are presented in Table 3. In these seven cases the imaging findings were considered to be congruent with the final cause of death at full autopsy as determined during the consensus meeting (Table 4). Of these cases the cause of death was cardiovascular in 2 (out of a total of 11 cardiovascular cases, 18.1%; Figs. 2 and 3), infectious disease in 1 case (out of a total of 16 infectious disease cases, 6.3%) and digestive tract in 4 cases (out of a total of 4 digestive tract cases, 100%; Figs. 4 and 5). In 7/54 (12.9%) children, additional imaging findings (e.g., rib fractures, an intrathoracic neuroblastoma and a congenital pulmonary adenomatoid malformation) were found and were clinically relevant but did not lead to a definitive cause of death. In the remaining 40/54 (74.1%) cases, postmortem CT did not add to the overall diagnostic workup.

**Table 3:** Postmortem computed tomography (PMCT) with relevant findings and congruent cause of death compared to autopsy

Case	Sex^1^	Age^2^	Imaging finding		Cause of death
			Digital radiography^3^	PMCT^4^	
1	F	17y/2m	Normal findings	Hematopericardium and aortic aneurysm	Hematopericardium and aortic aneurysm resulting from Loews-Dietz syndrome
2	M	0m/5d	Rib asymmetry	Tracheal right upper lobe bronchus, AVSD	Unbalanced AVSD
3	F	1y/4m	Normal findings	Right sided incarcerated inguinal herniation	Systemic infection resulting from incarcerated inguinal herniation
4	M	1y/0m	Normal findings	Small bowel dilation based on internal herniation or adhesion ileus	Adhesion ileus
5	F	6y/11m	Normal findings	Internal small bowel herniation	Adhesion ileus
6	F	2y/0m	Normal findings	Small bowel dilation based on internal herniation or adhesion ileus	Adhesion ileus
7	M	4y/7m	Normal findings	Ileocolic intussusception	Ileocolic intussusception resulting from a Meckel diverticulum

*AVSD* atrioventricular septal defect, *F* female, *M* male

**Table 4:** Postmortem CT versus autopsy in children with a conclusive cause of death based on full autopsy

		Conventional autopsy diagnosis		
		Definitive	Inconclusive	
Postmortem CT	Matching autopsy	7	16	23
	Non-matching autopsy	31	0	31
		38	16

## Discussion

In the last decades there has been an increasing interest in the use of postmortem radiology; initially the focus was on adults, often in forensic radiology [12–14]. However in the last several years there has been a strong increase in interest in postmortem radiology in children [15]. This has been recognised within the European Society of Paediatric Radiology, and in 2015 the society initiated a task force on postmortem radiology [16].

Our study shows that postmortem CT detected the cause of death in a minority of cases (12.9%) in a group of children who unexpectedly died of an apparent natural cause and in whom a cause of death was found. Based on the findings of this study, an autopsy could have been avoided in some children based on CT findings. In the majority of cases (74.1%) postmortem CT was not of added value in diagnosing the cause of death. One important explanation for this relatively low yield of postmortem CT could be the case mix, with a high rate of infection and cardiovascular causes of death. Postmortem CT did not yield clinically relevant findings in the majority of these cases.

The four excluded cases in whom an indication for a non-natural cause of death was found, although excluded from the final analysis, show that postmortem CT might, however, have a significant outcome on the final diagnosis of the cause of death in children and where findings might lead to legal investigations.

In the NODO procedure postmortem CT was used instead of postmortem MRI during this study. The main reason for this was the fact that the postmortem CT was initially planned to screen for potential non-natural causes of death, in which case the NODO procedure has to be aborted. For this purpose postmortem CT is sufficient because it rules out, for example, intracranial and thoracoabominal haemorrhages and fractures. The second reason is, given the high demand for MRI studies, access to MRI scanners is limited, particularly during regular office hours. In the current literature there is overwhelming evidence that in foetuses (which were excluded from the NODO procedure) and neonates, postmortem MRI is superior to postmortem CT [8, 17].

The Magnetic Resonance Imaging Autopsy Study (MaRIAS) from London showed that a minimal invasive autopsy consisting of a postmortem MRI in combination with postmortem blood sampling via a needle puncture had a high concordance rate, ranging from 53.6% to 81%, with the conventional autopsy [8]. It is also of interest to note that in the MaRIAS the concordance rate dropped significantly with increasing age, albeit with a much smaller number of older children. A smaller sub-study consisting of 82 cases from the MaRIAS cohort directly compared postmortem MRI versus postmortem CT [17]. This study showed that although postmortem CT was significantly less accurate compared to postmortem MRI in foetuses younger than the gestational age of 24 weeks, the modalities’ performance was not significantly different for foetuses older than 24 weeks’ gestational age, neonates and children.

In a study by Proisy et al. [5] conventional autopsy revealed a cause of death in 18 of 47 cases (38.3%). In 16 of these 18 cases postmortem CT showed concordant findings [5]. In a paediatric forensic case series Sieswerda-Hoogendoorn et al. [7] showed that the case mix is an important factor in predicting the sensitivity and specificity of postmortem CT. These studies show that, especially in older children, data are still lacking with respect to the accuracy of postmortem CT and postmortem MRI. Albeit in a small sample size, our study showed that in cases where the cause of death was related to the gastrointestinal tract the sensitivity was 100%, and for cardiovascular causes this was 16.7%. For all other causes of death the sensitivity was lower (decreasing to 0% for endocrinological causes, for example). The single case where infection was diagnosed as cause of death on postmortem CT was a case of an incarcerated inguinal hernia, with massive air in all vessels, so one could argue that this case actually should be considered as a gastrointestinal cause of death. Our study is one of the larger paediatric postmortem CT studies in non-forensic cases, yet in order to assess the true value of postmortem CT larger prospective multicentre studies are needed.

A drawback of our retrospective nested cohort study is the fact that not all children who were eligible, despite the fact that it is mandatory by law, were indeed referred for the NODO procedure. The mortality data of 2013, which are presented in the introduction, are the best available; more specific data for Dutch inhabitants younger than 18 years are not available. We also do not know the proportion of these children eligible for the NODO procedure. Based on the data from Statistics Netherlands, we estimate that close to 200 cases per year could have been included. There were significant differences across the Netherlands, where some areas didn’t submit a single case for the NODO procedure. The reason for this is unknown. It might — despite education and widespread information of the involved medical specialties — be related to recent implementation of the procedure in the Netherlands. We also cannot exclude the risk that selection bias has occurred because we do not have access to the death certificates of all children in the Netherlands. However we have good contacts in the field of forensic medicine and as far as we can assess there doesn’t seem to be a significant selection bias in our population. A second drawback is the fact that in nine cases no consent for autopsy was obtained. We therefore excluded these patients from our study; in one of these cases anterior rib fractures resulting from resuscitation were found, and in one case retroperitoneal and perirenal free air, possibly related to an infectious cause, was found. A final drawback is the fact that we could not evaluate the interobserver variance among the involved radiologists. The number of cases and their often distinctive findings would make it almost impossible to truly assess the interobserver variance. On the other hand, the study reflects a real-life situation where in general a single radiologist reviews the postmortem CT and interprets the findings.

In order for postmortem imaging to have a real impact in clinical practice both radiologists and pathologists need to work together or acquire a new skill set. In general, where radiologists lack pathological knowledge, pathologists lack radiologic knowledge; therefore during the NODO procedure, the ideal situation is a full child death review where all involved specialties discuss the cases. If this cannot be achieved, combined training in radiology and pathology could be a viable option [18–20].

## Conclusion

Although based on a relatively small study population, our study shows that in a proportion of children postmortem CT can have a significant impact on the diagnostic process. In this study postmortem CT allowed for the detection of potential cases of non-natural cause of death among a group in which natural death was expected. We feel that in a limited number of cases, especially in those with a cause of death related to the digestive tract, the combination of the clinical history and postmortem CT findings were sufficient to come to a cause of death and therefore might have obviated the need for an autopsy.

### Electronic supplementary material

Fig A1 Flowchart of the Dutch NODO procedure. *CoD* Cause of death (GIF 79 kb)

High Resolution (TIFF 763 kb)

## Appendix A: Legal framework of the Dutch NODO Procedure

To understand the full extent of the NODO procedure it is necessary to give some explication about the general procedure in postmortem investigation in the Netherlands. The Dutch procedure is as follows: Every deceased gets an external investigation by either the general practitioner at home, an attending medical doctor in a hospital or the forensics physician. The general practitioner and the attending medical doctor are allowed to handle deaths by natural cause. In the Netherlands the (exact) cause of death does not have to be clear to the general practitioner or the attending medical doctor in order for either to be convinced the death is from a natural cause. However when the general practitioner or the attending medical doctor is not convinced of death by a natural cause, he or she calls in the help of a forensic physician. The forensic physician does an external postmortem investigation. When further investigation is needed, a forensic autopsy is performed by a forensic pathologist. In the Netherlands all forensic autopsies take place at the department of forensic medicine of the Netherlands Forensic Institute, the Hague. In the Netherlands about 350 forensic autopsies are performed annually; of these approximately 30–40 are paediatric autopsies.

The doctor who carries out the external postmortem investigation fills in two forms: the so-called A and B forms. The A-form is for death by natural cause and goes to the registry office of the municipality. In case of a non-natural cause of death, the A-form is not filled in. Either way, the B-form is sent to Statistics Netherlands for census data in both natural and non-natural causes of death.

For children, a special procedure can be applied. Article 10a subsection 1 of the Burial Act obligates the general practitioner or the attending medical doctor to report a death of a minor under all circumstances to the forensic physician. The forensic physician gives the general practitioner or the attending medical doctor advice about how to handle the case. When there are signs of an accident, a suicide or a crime, the forensic physician takes over the procedure from the general practitioner or the attending medical doctor, and after an external investigation he reports to the public prosecutor. In cases in which a crime is suspected, the public prosecutor orders an internal forensic postmortem investigation.

When the cause of death is clear and appears to be a disease, the general practitioner or the attending medical doctor can fill in the death certificate (forms A and B). When the cause of death is unclear the forensic physician can start a further investigation into the death (NODO, Nader Onderzoek Doodsoorzaak Overleden minderjarige). Further investigation into the cause of death of children is mandated by Article 10a subsection 2 of the Burial Act) (Fig. A1, supplementary online). The NODO procedure is carried out by a specially trained forensic physician and paediatrician. This stepwise investigation goes as follows:

1. The paediatrician and forensic physician obtain a full clinical history from the parents/caretakers.
2. They assemble medical information from other caregivers, who have the obligation to give the information instantly (Article 10a subsection 3). This implies that for this specific category of patients the physician–patient privilege does not apply.
3. A paediatric radiologist reviews the postmortem CT.
4. A skeletal survey is conducted for every child younger than 4 years. This is reviewed by a paediatric radiologist. The skeletal survey should be performed according to the guidelines as set by the Royal College of Radiologists and the Royal College of Paediatrics and Child Health [10]. The aim of the skeletal survey is to detect occult fractures that might indicate a non-natural cause of death from skeletal anomalies, which might indicate a presence of a yet unknown underlying disease or syndrome.
5. Blood, urine, liquor are tested for chemistry, infectious and hereditary diseases and intoxication.
6. Skin biopsy is obtained for hereditary diseases.
7. Autopsy is performed.

After every step of the NODO procedure the team, under the lead of the forensic physician and paediatrician, decides whether the cause of death is clarified. The final cause of death is determined in an audit of specialists.

Until July 2016, the first six steps of the NODO procedure could be done without the consent of the parents. For the last step, the internal postmortem investigation, the consent of the parents was needed (Article 74 of the Burial Act). When the parents did not consent, the case could be presented to a judge. The judge could then consent to the postmortem investigation. Eventually the NODO procedure was being carried out at two centres, i.e. Emma Children’s Hospital – Academic Medical Center Amsterdam and the Wilhelmina Children’s Hospital – University Medical Centre Utrecht.

As of July 1, 2016, consent for every step in the NODO procedure was made mandatory. Now when no consent is given, the procedure is not started. Two other changes were made: the procedure is now carried out at all academic centres in the Netherlands, and postmortem CT has been replaced in all children younger than 2 years by postmortem MRI, and the radiologist and paediatrician can choose between postmortem CT and MRI in children ages 2–5 years. For patients ages 5 years and older, postmortem CT is advised because there is no evidence that either of these two techniques is superior and postmortem CT is easier, faster and cheaper to perform.
