# Supplementary figures and images for: The value of postmortem computed tomography in paediatric natural cause of death: a Dutch observational study
Source: Pediatr Radiol. 2017 Jul 5;47(11):1514–22. doi: 10.1007/s00247-017-3911-0 (PMC5608837; doi:10.1007/s00247-017-3911-0)

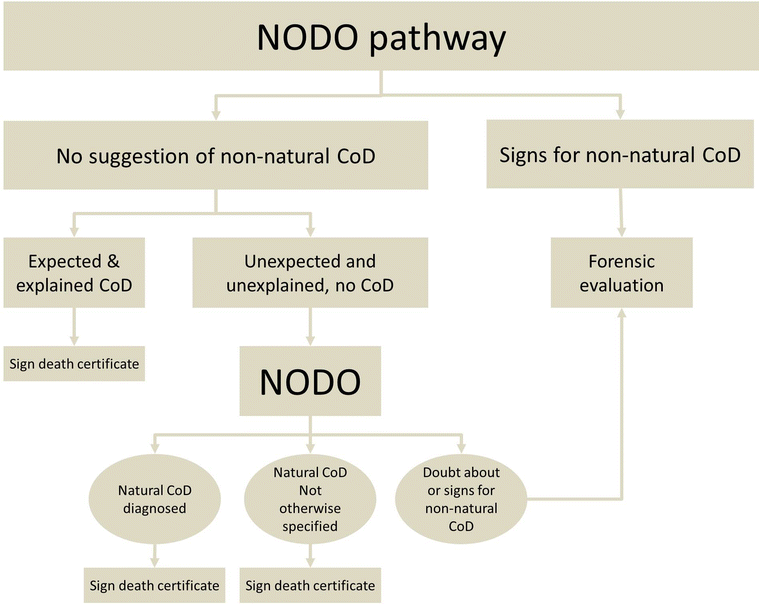

Supplement: Supplementary file 1 — Flowchart of the Dutch NODO procedure. CoD Cause of death (GIF 79 kb) [file 247_2017_3911_Fig6_ESM.gif]

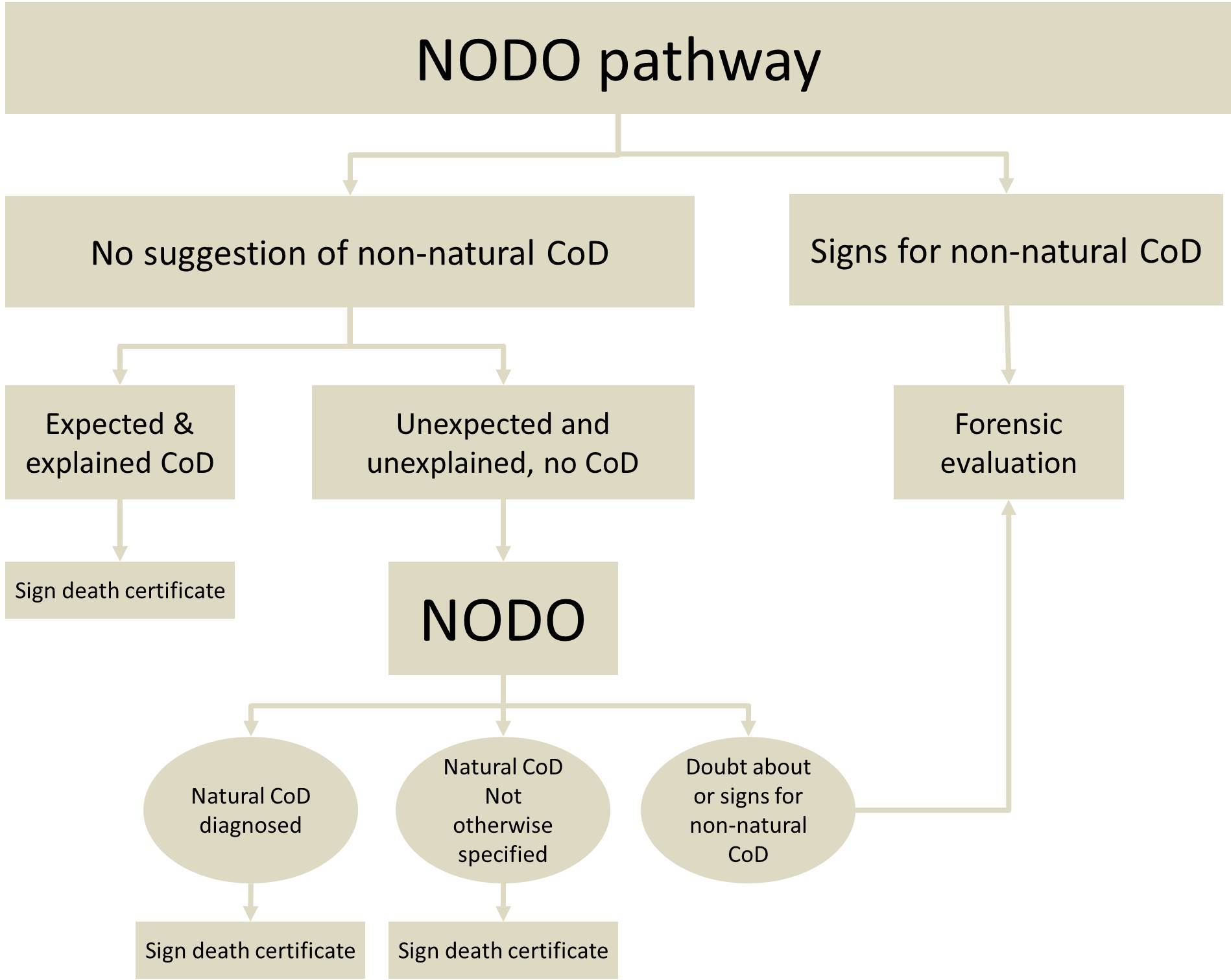

Supplement: Supplementary file 2 — High Resolution (TIFF 763 kb) [file 247_2017_3911_MOESM1_ESM.tif]
